# Genesis of charge orders in high temperature superconductors

**DOI:** 10.1038/srep18675

**Published:** 2016-01-06

**Authors:** Wei-Lin Tu, Ting-Kuo Lee

**Affiliations:** 1 Department of Physics, National Taiwan University, Daan Taipei 10617, Taiwan; 2 Institute of Physics, Academia Sinica, Nankang Taipei 11529, Taiwan

## Abstract

One of the most puzzling facts about cuprate high-temperature superconductors in the lightly doped regime is the coexistence of uniform superconductivity and/or antiferromagnetism with many low-energy charge-ordered states in a unidirectional charge density wave or a bidirectional checkerboard structure. Recent experiments have discovered that these charge density waves exhibit different symmetries in their intra-unit-cell form factors for different cuprate families. Using a renormalized mean-field theory for a well-known, strongly correlated model of cuprates, we obtain a number of charge-ordered states with nearly degenerate energies without invoking special features of the Fermi surface. All of these self-consistent solutions have a pair density wave intertwined with a charge density wave and sometimes a spin density wave. Most of these states vanish in the underdoped regime, except for one with a large d-form factor that vanishes at approximately 19% doping of the holes, as reported by experiments. Furthermore, these states could be modified to have a global superconducting order, with a nodal-like density of states at low energy.

Ever since the discovery of the high-Tc superconductivity, many low-energy charge-ordered states in the cuprate have been discovered. Neutron scattering experiments^1^ first emphasised the doping dependence of incommensurate magnetic peaks associated with unidirectional magnetic patterns or stripes. Later, soft X-ray scattering^2^ also confirmed the presence of charge orders with these stripes. However, these experiments were performed on the 214 (*La*_2−*x*_*Sr*_*x*_*CuO*_4_) cuprate family. For other cuprate families, the evidence for bond-centred unidirectional domains was found via scanning tunneling spectroscopy^3^,^4^. The charge density wave (CDW) order was also found to be induced by the external magnetic field^5^. Recently, more results regrading charge-ordered states^6^,^7^,^8^,^9^,^10^, and electron-doped cuprates^11^ have been reported. The periods of these CDW and their doping dependence are quite different for different cuprate families^10^. In addition to the unidirectional stripe pattern, some experiments have also reported the possible existence of a bidirectional charge-ordered checkerboard pattern^12^,^13^. The unidirectional charge-ordered states or stripes were found to have a dominant d-like symmetry for the intra-unit-cell form factor, measured on the two oxygen sites by using the resonant elastic x-ray scattering method^14^,^15^ and via scanning tunneling spectroscopy (STS)^16^. However, different families seem to prefer different symmetries^14^,^15^. In the STS experiments^17^, the density waves disappeared above 19% hole doping. Furthermore, the observation of these CDW states having nodal-like local density of states (LDOS) at low energy but strong spatial variation at high energy in STS^3^ strongly implies a new unconventional superconducting state.

The existence of these great varieties of charge-ordered states has created a great debate regarding whether the strong coupling Hubbard model or the *t* − *J* model^18^ is the proper basic Hamiltonian to describe the cuprates. Many believe that these states “compete” with the superconductivity^19^ and that their origin may reveal the fundamental understanding of the mechanism of high superconducting temperatures in cuprates. The recent detection of the d-form factor at an oxygen site instead of at a Cu site^14^,^15^,^16^ also raises the question about the suitability of the effective one-band Hubbard or *t* − *J* model and the validity of replacing the oxygen hole with a Zhang-Rice singlet^20^, which effectively supports a simpler one-band model with Cu only. Allais *et al.*^21^ proposed that the d-symmetry of these form factors, referred to as bond orders^22^,^23^ because they are measured between the nearest neighbour Cu bonds, arise from the strong correlation but without other intertwined orders. Furthermore, there are also doubts regarding whether a strong correlation is present or even needed to understand of the superconducting mechanism^24^. However, the complexities of the phase diagram and some recent theoretical works have indicated the possibility of a new phase of matter, i.e., the pair density wave (PDW)^25^,^26^,^27^,^28^, as discussed in detail in a recent review article^25^. The new states are considered to have intertwined orders of PDW and CDW or spin density waves (SDW)^25^.

For quite some time, various calculations^29^,^30^,^31^,^32^,^33^,^34^,^35^,^36^,^37^,^38^,^39^ on the Hubbard and *t* − *J* type models have revealed low-energy intertwined states appearing as stripes or bidirectional charge-ordered states, such as checkerboard (CB). However, these works usually involved different approximations and parameters, which often resulted in different types of charge-ordered patterns, and these studies were mostly concentrated at a hole concentration of 1/8, which is the most notable concentration in early experiments. Hence, it is not clear if these results were the consequence of the invoked assumption or the approximation used, or if they are a generic results in the phase diagrams of cuprates. There were attempts to produce these CDWs or PDWs using a different approach, such as using a mean field theory to study a *t* − *J*-like model but taking the strong correlation as only a renormalization effect of dispersion^22^,^23^,^40^,^41^. A spin-fluctuation mediated mechanism to produce these states was also proposed for the spin-fermion model^42^. Recently, a novel mechanism of PDW was proposed, i.e., Amperean pairing^28^, by using the gauge theory formulation of the resonating-valence-bond picture. In most of these approaches, the wave vectors or periods of the density waves are related to special features of the Fermi surface, including nesting, hot spots or regions with large density of states. However, the opposite doping dependence of CDW periods, observed for 214 and 123 (*YBa*_2_*Cu*_3_*O*_6+*δ*_) compounds^10^, makes the Fermi surface scenario worrisome.

Amid all this confusion, recent numerical progress achieved by using the infinite projected entangled-pair states (iPEPS) method^43^, has provided us with a new clue. It was found that the *t* − *J* model has several stripe states, with nearly degenerate energy as the uniform state and, with coexistent superconductivity and antiferromagnetism. When the number of variational parameters is extrapolated to infinity, the authors concluded that the anti-phase stripe, which has no net pairing, has slightly higher energy than the in-phase stripe with a net pairing, which in turn, also has slightly higher energy than the uniform state. This result is very consistent with the result of variational Monte Carlo calculations^29^ based on the concept of the resonating-valence-bond picture^18^. Furthermore, the result is also consistent with that of renormalized mean-field theory by using a generalised Gutzwiller approximation (GWA)^44^ to treat the projection operator in the *t* − *J* model^30^,^45^. Hence, the result provides strong support to more carefully examine the low energy states of the *t* − *J* model with the variational approach using GWA.

Here, we report our findings from a much more extensive examination of the renormalized mean-field theory prediction using the GWA for the hole-doped *t* − *J* model. We find many unidirectional and bidirectional charge-ordered states with nearly degenerate energies as the uniform state, especially in the lightly doped regime; thus, it is a much more general phenomenon than previously thought. All of these states have intertwined orders of PDW, CDW and/or SDW. One of the CDW states, denoted as AP-CDW, reveals a bond order pattern with a much larger d-form factor than *s*′ symmetry, as found in the experiment^16^ with BSCCO (*Bi*_2_*Sr*_2_*CaCu*_2_*O*_8+*x*_) and NaCCOC (*Ca*_2−*x*_*Na*_*x*_*CuO*_2_*Cl*_2_). Furthermore, just as in the experiment^17^, it vanishes beyond 19% hole doping. However, not all these charge-ordered states have a dominant *d*-form factor. For example, a different CDW intertwined with SDW and PDW, which is the familiar stripe reported long ago for 214^1^,^2^,^29^,^31^,^36^, has a larger *s*′ form factor, as reported in the experiment^15^. We further show that this AP-CDW state could be easily altered to become a superconducting state with a global d-wave pairing symmetry, while locally, each bond does not have the perfect d-wave symmetry. Its spectra shows a large spatial variation at higher energies but with a d-wave nodal-like LDOS near zero energy as seen in the experiments^2^,^17^.

## Results and Discussions

As mentioned above, the variational approach has been quite effective at capturing the physics of the strong correlation present in the *t* − *J* model. By using GWA, we can replace the strong constraint of forbidding the double occupancy of two holes on the same site in the variational wave function using Gutzwiller factors^32^,^33^,^44^,^45^. Then, one can use just mean field theory to find the various low energy states. Details about the calculation are discussed in the Methods section.

In our mean field theory, there are four variational order parameters. Besides the hole density *δ*_*i*_, the local spin moment 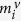 provides the antiferromagnetic correlation, the pair field 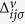 represents the local electron pairing order, and bond order 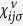 is just the kinetic hopping term, where *i* is a site position and *ij* is the nearest neighbour bond. An iterative method is used to self-consistently solve the mean-field Hamiltonian *H*_*MF*_ (Eq. (S7) in the Supplementary Material (SM)) for all the parameters, of which there could be more than 60. The convergence is achieved for every order parameter if its value changes by less than 10^−3^ between successive iterations. All the calculations are performed on a 16 by 16 square lattice. To obtain various charge orders, specific patterns of *δ*_*i*_, 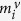, and 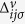 are input as initial values. The bond orders 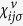 are always initially assumed to be uniform. In most cases, we will obtain only uniform solutions such as the d-wave superconducting (dSC) state and/or coexistent antiferromagnetic (dSC-AFM) state, but sometimes the states with charge-ordered patterns are found as a self-consistent solution.

### Charge-ordered Patterns

In addition to the two uniform solutions of a dSC state and a dSC-AFM state, there are many non-uniform charge-ordered states. For simplicity, we shall first present those charge-ordered states with a period of four lattice spaces (4*a*_0_), as listed in Table 1. Both the pair field 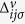 and the spin moment 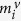 could have positive and negative values. It turns out that if there is a SDW or a bidirectional spin CB (sCB) present, then it always has a period of 8*a*_0_, with two domains of size 4*a*_0_ with opposite antiferromagnetic directions joining together. The pair field has more choices. It could always be positive, with all of its x-bond pair field being positive and y-bond pair field being negative: thus, it would have a net total non-zero pair field. This is called an in-phase (IP) state, with a period of 4*a*_0_. However, just like the spin moment, the pair field could also have two domains with opposite signs and a domain wall in between: this state is known as the anti-phase (AP) state, with a period of 8*a*_0_. Thus, we could have four possible states for each unidirectional CDW or bidirectional charge CB (cCB), as we either have an IP or AP pair field with or without SDW. However, we only have three such states in Table 1 because we cannot find a solution with an IP pair field and CDW only. This result is due to the choice of the commensurate period being 4*a*_0_. Later, we will show a state with a net pairing order or IP pairing state and CDW, which occurs if we do not require solutions to be commensurate with the lattice.

**Figure 1:**
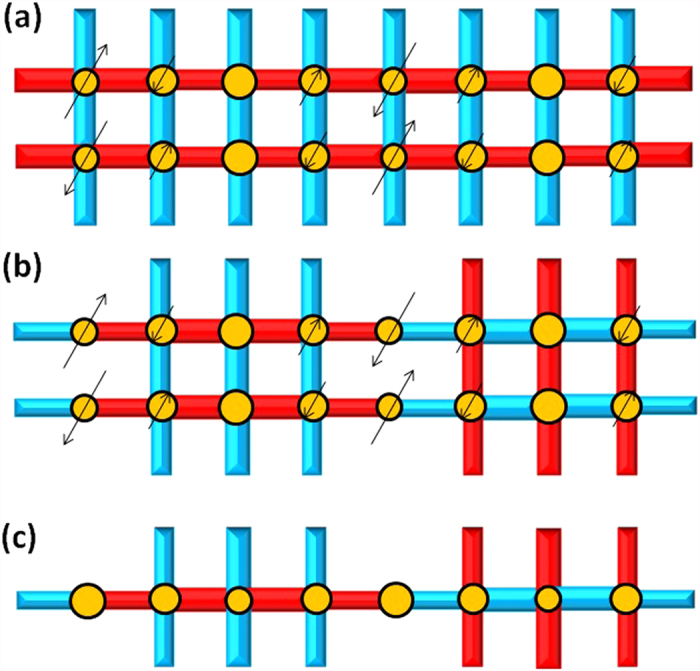
Schematic illustration of modulations for stripe like patterns. (**a**) IP-CDW-SDW (**b**) AP-CDW-SDW (**c**) AP-CDW respectively. Size of the circle represents the hole density. The width of the bond around each site represents the amplitude of pairing Δ(Δ = ∑_*σ*_Δ_*σ*_) and sign is positive (negative) for red (cyan). The size of black arrows represents the spin moment. The average hole density is about 0.1.

Figure 1 shows a schematic illustration of the modulations of the pair field, charge density and spin moment for the three stripes with hole concentration of 0.1. The magnitude of the pair field is proportional to the width of the bond; red (cyan) denotes positive (negative) value. The size of the arrow is proportional to the spin moment, and the size of the circle represents the hole density. A similar figure for the three bidirectional CB patterns is shown in Figure S1 in SM. There is one domain wall corresponding to the vanishing spin moment for IP-CDW-SDW in Fig. 1a or the vanishing pair field for AP-CDW in Fig. 1c. Both domain walls are present for the AP-CDW-SDW states in Fig. 1b. The hole density is always maximum at the domain wall with the vanishing spin moment. However, if there is no SDW, such as the AP-CDW stripe in Fig. 1c, then the hole density is maximum at the domain wall with the vanishing pair field. This finding is different from previous work without including the renormalized chemical potential^37^.

**Figure 2:**
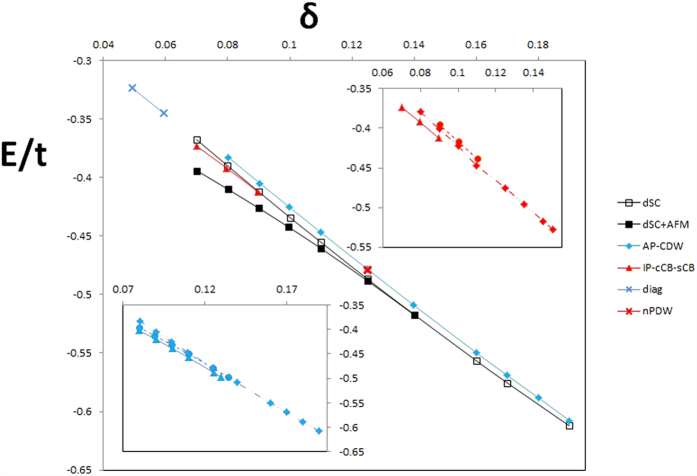
Energy per site as a function of hole concentration. Six states are shown in the main figure with notations defined in Table 2. The lower (upper) inset is for stripe (CB) patterns. Blue triangles, circles, and diamonds are for IP-CDW-SDW, AP-CDW-SDW, and AP-CDW respectively. And red triangles, circles and diamonds are for IP-cCB-sCB, AP-cCB-sCB, and AP-cCB respectively.

Figure 2 shows energies as a function of hole concentration for all the states listed in Table 1. The three unidirectional states are shown in the lower inset with blue triangles, circles, and diamonds representing IP-CDW-SDW, AP-CDW-SDW, and AP-CDW, respectively. The three CB states are shown in the upper inset with red triangles, circles and diamonds representing IP-cCB-sCB, AP-cCB-sCB, and AP-cCB, respectively. Unless specifically mentioned, we only report site-centred results. Bond-centred solutions have essentially the same energies. The same results for the three CDW states were also reported in ref. 30 at a 1/8 hole concentration. These mean-field GWA results are quite consistent with the numerical Monte Carlo result^29^, which revealed that the uniform state has the lowest energy, followed by the in-phase stripe, and that the energy of the anti-phase stripe is slightly above that of both of them. However, the small energy differences are insignificant compared to the result of iPEPS^43^, which showed the same ordering of states but with essentially degenerate energies.

At approximately 12% doping in Fig. 2, the spin moment becomes smaller, and the uniform dSC-AFM state merges into the dSC state. The difference from the original work of Ogata and Himeda^32^,^33^, in which the spin moment vanished at 10% doping, is due to the simplified Gutzwiller factors used in Eq. (4). All the magnetic states, such as SDW and sCB, vanish at approximately 12% doping. The most surprising and important result shown in Fig. 2 is that in addition to the uniform dSC state, the AP-CDW state is most stable for a large doping range, from 0.08 to 0.18. The AP-cCB state also extends a little bit beyond the antiferromagnetic region. We only find the diagonal stripe state up to 6% doping. Another pattern that seems to be limited to small doping is IP-cCB-sCB, which is only found at doping less than 0.1. The general locations of these CB states in Fig. 2 are consistent with experimental observations that CB are seen more often at low doping^12^,^13^. Because the Gutzwiller factor 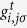 in Eq. (4) is proportional to the hole density at the site, we expect the kinetic energy to be maximum at the domain wall (Fig. 1c), as shown in Table 2. Table 2 lists the values of hole density, the magnitude of the pairing order parameter and the kinetic energy *K* at each site, which are calculated by averaging the four nearest neighbour hopping amplitudes for AP-CDW at a 1/8 hole concentration. The kinetic energy and pairing order are calculated from the variational parameters 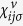 and 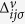 respectively, by using Eq. (S9) in SM. Similar tables for other stripes and CB patterns are presented in Tables S1 and S2 in the SM.

**Figure 3:**
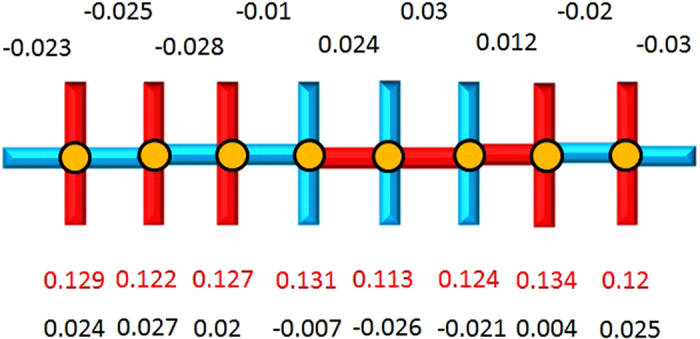
Schematic illustration of modulations for nPDW stripe. The numbers in red denote the hole dnesity at each site while the numbers in black below them represent the pairing amplitude in y direction. The rest numbers above the figure stand for the pairing amplitude in x direction. Here our pairing amplitudes denote (〈*c_i↑_c_j↓_*〉). Note that in this figure neither the size of circles nor the width of bonds represent amplitudes. The hole concentration is 0.125.

**Figure 4:**
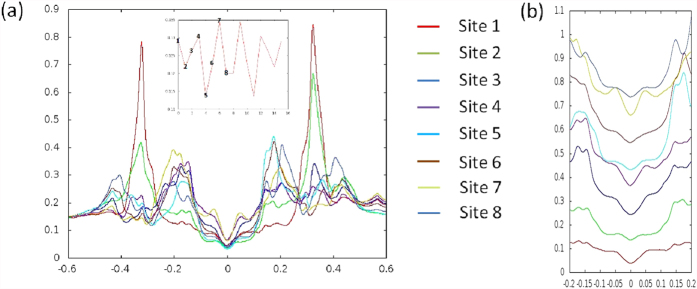
(**a**) LDOS at 8 sites plotted from energy 0.6t to −0.6t. The inset shows hole density along the modulation direction of the nPDW stripe and (**b**) from 0.2t to −0.2t but shifted vertically for clarity.

The red cross in Fig. 2 at the 1/8 hole concentration is the energy of a solution as a result of relaxing the requirement to have a commensurate 4*a*_0_ period for the AP-CDW state. To alleviate the difficulty of considering incommensurate solutions in a finite lattice calculation, we allow the state to have more than one single modulation period. In Fig. 3, the hole density, listed as the red numbers below the pattern, along with the magnitude of the pairing order parameter for both x and y bonds, listed in the top and bottom rows, are plotted along the direction of the modulation for a complex bond-centred stripe of length 16*a*_0_. It is very similar to the AP-CDW state. However, there is a remaining net constant d-wave pairing, with the system average Δ_*x*_ = −0.0056 and Δ_*y*_ = 0.0057. This mixture of the AP-CDW stripe with a small constant uniform pairing will produce a d-wave nodal-like LDOS in addition to a PDW; hence, we have a nodal PDW or nPDW. There are several important results associated with the nPDW. Figure 3 shows that the hole density is indeed maximum at the domain walls near sites 4,7,10 and 13. The maximum amplitude of pairing order Δ is about 0.03, which is roughly the same as adding the net pairing amplitude to that of the AP-CDW stripe in Table 2. It is most gratifying to observe that the d-wave pairing is globally maintained, although we have no way of controlling it during the iteration, with variables changing independently on each site. Contrary to the pure AP-CDW state without a net pairing, this state has a d-wave nodal spectrum at low energy, hence a nodal-like LDOS. In Fig. 4a, the LDOS of this stripe at 8 sites is plotted as a function of energy. The positions of these 8 sites are indicated in the inset of Fig. 4a. The detailed LDOS at low energy is shown in Fig. 4b. The large spatial variation of LDOS at high energies but always with a d-wave node near zero energy is quite consistent with the STM results in ref. 3. We have obtained this result by using a lattice of 16 × 16 supercells; please see the SM for details.

A special feature of all these charge-ordered states is the large variation of the Gutzwiller factors from site to site. The values could change between nearest neighbours by a factor of 2 to 3. This unique property of strong correlated systems originates from the dependence on local hole density in the Gutzwiller factor, which is 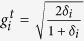, when we do not consider magnetic moments. This dependence on *δ*_*i*_ is the consequence of being a Mott insulator when there are no doped holes. A slight variation of the hole density *δ*_*i*_ will cause a large change in 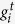; in fact, 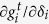 is proportional to 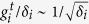. This factor dominates in the renormalized local chemical potential defined in Eq. (S6) when hole concentration is small. Thus, 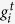 is no longer a purely passive renormalization factor; now, it could alter the local chemical potential greatly and induce non-uniform charge orders. Although the factor associated with spin, 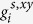 in Eq. (4), is smaller, it also contributes to the local chemical potential. The strong susceptibility to the variation of local hole density makes a uniform state unstable amidst inherent or extrinsic charge fluctuations. This effect is clearly more prominent in the lightly hole-doped regime, as demonstrated by the greater variety of charge-ordered states in the underdoped regime in Fig. 2. Another important effect of the Gutzwiller factor is that it introduces nonlinearity into the Bogoliubov-deGenne (BdG) equations (Eq. (S4)–(S6)), which can produce quite unexpected solutions.

### Bond Order

**Figure 5:**
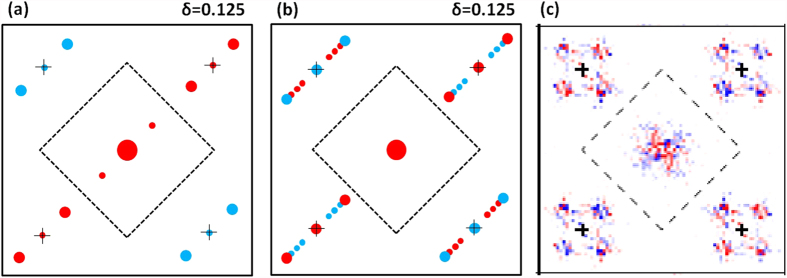
Schematic illustration of the Fourier transform of bond orders of (a) AP-CDW state and (b) the nPDW stripe in a lattice of 16*a*_0_ * 16*a*_0_. “+” signs are at the four reciprocal lattice vectors (±2*π*/*a*_0_, 0) and (0, ±2*π*/*a*_0_) and their nearby medium size dots are shifted from them by (±*π*/2*a*_0_, 0). The large dot at center is *Q* = (0, 0) and has two red small dots nearby at (±*π*/2*a*_0_, 0). The inner dotted square is the boundary of first Brillouin zone. (**c**) is copied from Fig. 3G of the STS work of Fujita *et al.*^16^. It shows the sum of real part of Fourier transform values of tunneling currents measured at *O*_*x*_ and *O*_*y*_ sites. Unlike (**a**,**b**) that only has one domain of density modulation in the x direction, this sample has two domains with both x and y direction modulations.

So far, we have only discussed the pair field, hole density and spin moment; now, we shall consider more carefully the bond order 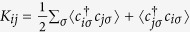. The value of one-half in front of the summation is for averaging because there are two hopping terms for each bond. Now, it can be calculated by using the BdG solution and the Gutzwiller factor, i.e., 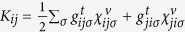. Following the definition of bond order by Sachdev and collaborators^22^,^23^,^40^ and Fujita *et al.*^16^, by associating 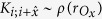, the tunneling current measured at the x-bond oxygen site can be obtained, similarly for the y-bond oxygen. The Fourier transform of these two quantities gives us the intra-unit-cell form factor. The Fourier transform of the AP-CDW state with a hole concentration of 1/8 is schematically shown in Fig. 5a. The size of the dot represents the magnitude; red (blue) represents a positive (negative) value. Because this is a 4*a*_0_ stripe, in addition to values at *Q* = (0, 0) and reciprocal lattice vectors denoted by the “+” sign, the modulation wave vector is (±*π*/2*a*_0_, 0), and the vectors are shifted by the reciprocal lattice vectors. The peaks at (±*π*/2*a*_0_, 0) are determined by *A*_*S*′_, while those at (±3*π*/2*a*_0_, 0) and (±*π*/2*a*_0_, ±2*πa*_0_) are determined by *A*_*D*_. The ratio of *A*_*D*_ to *A*_*S*′_, or *d*/*s*′, is approximately 7.5 in this case. This ratio is quite special for the AP-CDW state. For the IP-CDW-SDW stripe, the ratio is actually less than one. The schematic plots of the Fourier transform of IP-CDW-SDW and AP-CDW-SDW stripes are shown in Figure S2a and S2b in the SM, respectively. For the AP-CDW-SDW stripe, *d*/*s*′ is approximately 1.2. The Fourier transform of the bond orders of the AP-cCB pattern is similar to that of AP-CDW with a dominant d-form factor, as discussed in the SM.

The nPDW stripe shown in Fig. 3 also has a large *d*-form factor with almost zero *s*′. The Fourier transform of its bond order is schematically shown in Fig. 5b. The size of the dot scales with the magnitude of the *d*-form factors, and red (blue) represents a positive (negative) value. The wave vector with a large amplitude is at 5*π*/8*a*_0_ or its period is approximately 3.2*a*_0_. This length is close to the separation between the domain walls of the pair field shown in Fig. 3. The presence of smaller peaks at several wave vectors shows a mixture of different periods in the stripe. This result is expected if we add a constant pairing order to the AP-CDW stripe.

Figure 5c is copied from Fig. 3G of the STS work of Fujita *et al.*^16^. It shows the sum of real part of Fourier transform values of tunneling currents measured at *O*_*x*_ and *O*_*y*_ sites. Just like Fig. 5a,b, The value at (±3*π*/2*a*_0_, 0) is larger than that at (±*π*/2*a*_0_, 0) and both have the same sign but opposite sign with respect to (±*π*/2*a*_0_, ±2*πa*_0_). In their sample there are two domains with density modulation in x and y directions, respectively.

**Figure 6:**
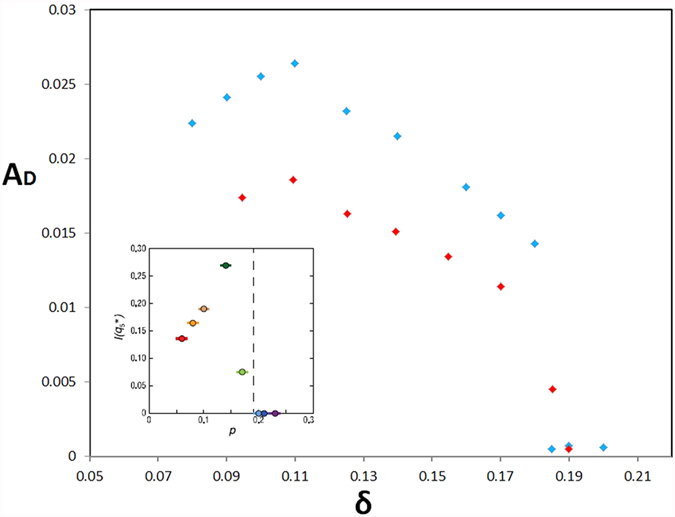
Magnitude of the d-form factor for the AP-CDW stripe as a function of doped hole concentration. Blue dots are for site-centered AP-CDW stripe and red ones for bond-centered AP-CDW. The inset is copied from Fig. 3G of the STS work of Fujita *et al.*^17^ showing the doping dependence of intensity of the modulation wave vector near (±3*π*/2*a*_0_, 0), which is associated with the density wave. This modulation vanishes at 19% hole concentration.

Another interesting result regarding the AP-CDW stripe is that its *d*-form factor actually vanishes at an approximately 19% hole concentration, as shown in Fig. 6 for both site-centred (blue dots) and bond-centred (red dots) solutions. We cannot find the AP-CDW solution beyond 18% doping. This outcome is in excellent agreement with the results reported by Fujita *et al.*^17^ in their Fig. 3G which is copied as the inset of Fig. 6. They measured the doping dependence of intensity of the modulation wave vector near (±3*π*/2*a*_0_, 0), which is associated with the density wave. The density wave disappeares at approximately 19% doping. Moreover, this 19% hole concentration is conspicuously close to the so-called quantum critical point^46^. We shall study this issue more in future work.

## Conclusion

The results reported above are all based upon the well-established renormalized mean-field theory^45^ and GWA^44^ for a well-studied *t* − *J* model. Although they do not provide extremely accurate numbers, as many sophisticated numerical methods do, our results show that they do capture the most important physics of the strong correlation. This strong correlation provides a site-dependent Gutzwiller renormalization that produces many exotic solutions of PDW stripes and/or CBs intertwined with modulations of charge density and/or spin density. These results show quantitative agreement with some of the key experiments^3^,^12^,^13^. Because site-renormalization is extremely local, the effect of the Fermi surface or wave vectors *k*_*F*_ is absent. Our model does not have the second or third neighbour hopping to provide a Fermi surface with nesting vectors or “hot spots”^22^,^40^,^46^. Thus, in our theory, there are no unique wave vectors for the charge density waves or CBs. Although we have mainly focused on the structures with a period of 4*a*_0_ so far, our preliminary study also finds charge-ordered states with periods of 5*a*_0_ and even 3*a*_0_. States with a longer period should be possible, and they could also have degenerate energies^34^,^43^. If we allow a pattern with multiple periods, such as the nPDW stripe shown in Figs 3 and 5b, we could have states with fractional or incommensurate periods. A detail study of all these will be conducted in the future, as well as a study of the effect of having values of *J*/*t* away from 0.3.

An important consequence of having all these charge-ordered states originating from the same Hamiltonian and physics is that these states are not the usual “competing states” we are familiar with. They do not stay in a deep local minima in the energy landscape. They are actually quite fragile and can easily evolve into each other, as we have already demonstrated with the nPDW stripe, which evolved from a mixture of AP-CDW and an uniform d-SC state. Other examples of the mixture of stripes listed in Table 1 can be easily constructed. For real cuprates, there are many other interactions in addition to our *t* and *J* that will alter the preferences of these states. For example, a weak electron lattice interaction could make the IP-CDW-SDW stripe much more stable against the dSC-AFM state^36^. Including special Fermi surface features could also enhance CDW for certain periods. However, none of these interactions are as important and necessary as the site renormalization due to strong Mott physics to produce these charge-ordered states. The effect of finite temperature will certainly bring in the entanglement of these states and much more complicated phenomena, such as pseudogap. Developing a method for generalising GWA to include the temperature effect remains as a big challenge.

## Methods

We introduce the *t* − *J* Hamiltonian^18^ on a square lattice of Cu by using


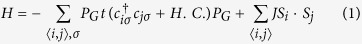


where nearest neighbour hopping *t*, as our energy unit, is set to 1, and *J* is set to 0.3. 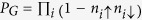 is the Gutzwiller projection operator, while 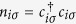 stands for the number operator for site i. Spin *σ* is equal to ±. *S*_*i*_ is the spin one-half operator at site i. The Fermi surface of the uniform state is quite simple, without nesting parts, and does not intersect with the magnetic Brillouin zone boundary, thus avoiding hot spots.

Following the idea of Gutzwiller^44^ and work of Himeda and Ogata^32^,^33^, we replace the projection operator (*P*_*G*_) with the Gutzwiller renormalization factors. The renormalized Hamiltonian now becomes


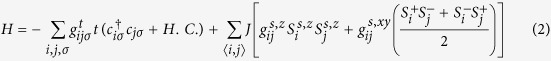


where 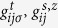, and 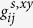 are the Gutzwiller factors, which are dependent on the values of local AF moment 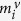, pair field 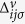, bond order 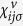, and hole density *δ*_*i*_:


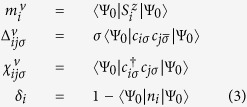


where 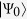 is the unprojected wavefunction. The superscript *v* is used to denote that these quantities are different from the real physical quantities for comparison with the experiments. Their relationship is given in Eq. (S9). As for the Gutzwiller factors, we follow the work of Yang *et al.*^30^; they used a slightly simplified version of Ogata and Himeda^32^,^33^, which was also used by Christensen *et al.*^34^. The factors are given as


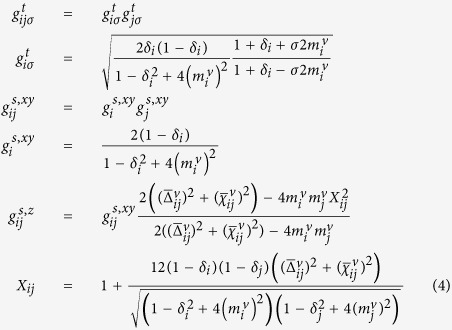


where 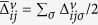 and 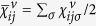. In the presence of antiferromagnetism, 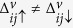. The derivation of the mean-field self-consistent equations is described in the SM.

## Additional Information

**How to cite this article**: Tu, W.-L. and Lee, T.-K. Genesis of charge orders in high temperature superconductors. *Sci. Rep.* **6**, 18675; doi: 10.1038/srep18675 (2016).

## Supplementary Material

Supplementary Information

## Figures and Tables

**Table 1:** Definition of various nearly degenerate states with respect to the intertwined orders: pair field, charge density, and spin moment.

	pair field	charge modulation	spin modulation
IP-CDW-SDW	in-phase	stripe	yes
AP-CDW-SDW	anti-phase	stripe	yes
AP-CDW	anti-phase	stripe	zero
IP-cCB-sCB	in-phase	checkerboard	yes
AP-cCB-sCB	anti-phase	checkerboard	yes
AP-cCB	anti-phase	checkerboard	zero
dSC	uniform	uniform	zero
dSC-AFM	uniform	uniform	uniform
diag	in-phase	stripe along (1, 1)	yes

Besides the two uniform solutions, d-wave superconducting (dSC) state and coexistent antiferromagnetic (dSC-AFM) state, all the states to be considered in this paper, unless specifically mentioned, have modulation period 4*a*_0_ for charge density and bond order. IP (AP) means the pair field is in-phase with period 4*a*_0_ (anti-phase with period 8*a*_0_). IP has a net pairing order and AP has none. SDW is the spin density wave with period 8*a*_0_. sCB (cCB) denotes the checkerboard pattern of spin (charge) and diag means the diagonal stripe which has in-phase pair field and spin modulation.

**Table 2:** Hole density and order parameters at each site for an AP-CDW stripe at 0.125 doping.

site number	1	2	3	4
*δ*_*i*_	0.1315	0.1256	0.1168	0.1256
Δ_*i*_	0	0.0194	0.0247	0.0194
*K*_*i*_	0.092	0.0866	0.0799	0.0866
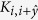	0.1151	0.0901	0.0625	0.0901
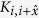	0.0688	0.0972	0.0972	0.0688

Δ_*i*_ is the average of pairing order of the four bonds at site *i*. *K*_*i*_ is the average kinetic energy at each site and 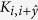 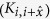 are the bond orders in the y (x) direction. These parameters are calculated according to Eq. (S9).
