# Supplementary material for: Genesis of charge orders in high temperature superconductors
Source: Sci Rep. 2016 Jan 6;6:18675. doi: 10.1038/srep18675 (PMC4702086; doi:10.1038/srep18675)
Supplement: Supplementary Information [file srep18675-s1.pdf]

Supplementary materials for: Genesis of charge orders in high temperature superconductors

Wei-Lin Tu[1,2,*] **and Ting-Kuo Lee**[2]

[1]Department of Physics, National Taiwan University, Daan Taipei 10617, Taiwan
[2]Institute of Physics, Academia Sinica, Nankang Taipei 11529, Taiwan
[*]stevedue19@gmail.com

ABSTRACT

In this supplementary materials we have further derived the mean-field self-consistent equations in **Methods** section of the main text. With the help of Bogoliubov-deGenne equation, we are able to get eigenfunctions and their corresponding eigenvalues for further utility, such as determining order parameters or calculating local density of states(LDOS). We also show similar tables and figures as those in the main text for other stripe or checkerboard patterns here.

Following the renormalized mean-field theory[1] by using the GWA as in the works of Yang *et al.*[2], we derive the formula we used for solving the BdG equations. After we replace the projection operator by the Gutzwiller factors and use the mean-field order parameters defined in Eq.(3), the energy of the renormalized Hamiltonian(Eq.(2)) becomes

$$\begin{aligned} E = \langle \Psi_0 \mid H \mid \Psi_0 \rangle = & -\sum_{i,j,\sigma} g^t_{ij\sigma} t(\chi^v_{ij\sigma} + H.C.) - \sum_{\langle i,j\rangle\sigma} J\Big(\frac{g^{s,z}_{ij}}{4} + \frac{g^{s,xy}_{ij}}{2}\frac{\Delta^{v*}_{ij\bar{\sigma}}}{\Delta^{v*}_{ij\sigma}}\Big)\Delta^{v*}_{ij\sigma}\Delta^v_{ij\sigma} \\ & - \sum_{\langle i,j\rangle\sigma} J\Big(\frac{g^{s,z}_{ij}}{4} + \frac{g^{s,xy}_{ij}}{2}\frac{\chi^{v*}_{ij\bar{\sigma}}}{\chi^{v*}_{ij\sigma}}\Big)\chi^{v*}_{ij\sigma}\chi^v_{ij\sigma} + \sum_{\langle i,j\rangle} g^{s,z}_{ij} J m^v_i m^v_j \end{aligned} \tag{S1}$$

Next we want to minimize the energy under two constraints: $\sum_i n_i = N_e$ and $\langle\Psi_0|\Psi_0\rangle = 1$. Thus our target function to be minimized is

$$W = \langle\Psi_0|H|\Psi_0\rangle - \lambda(\langle\Psi_0|\Psi_0\rangle - 1) - \mu\Big(\sum_i n_i - N_e\Big) \tag{S2}$$

The mean-field Hamiltonian now becomes

$$H_{MF} = \sum_{i,j,\sigma} \frac{\partial W}{\partial \chi^v_{ij\sigma}} c^\dagger_{i\sigma} c_{j\sigma} + H.C. + \sum_{\langle i,j\rangle\sigma} \frac{\partial W}{\partial \Delta^v_{ij\sigma}} \sigma c_{i\sigma} c_{j\bar{\sigma}} + H.C. + \sum_{i,\sigma} \frac{\partial W}{\partial n_{i\sigma}} n_{i\sigma} \tag{S3}$$

Eq.(S3) satisfies the Schr*ö*dinger equation $H_{MF}|\Psi_0\rangle = \lambda|\Psi_0\rangle$. The three derivatives are defined as

$$H_{ij\sigma} = \frac{\partial W}{\partial \chi^v_{ij\sigma}} = -J\Big(\frac{g^{s,z}_{ij}}{4} + \frac{g^{s,xy}_{ij}}{2}\frac{\chi^{v*}_{ij\bar{\sigma}}}{\chi^{v*}_{ij\sigma}}\Big)\chi^{v*}_{ij\sigma} - g^t_{ij\sigma} t + \frac{\partial W}{\partial g^{s,z}_{ij}}\frac{\partial g^{s,z}_{ij}}{\partial \chi^v_{ij\sigma}} \tag{S4}$$

$$D^*_{ij} = \frac{\partial W}{\partial \Delta^v_{ij\uparrow}} = -J\Big(\frac{g^{s,z}_{ij}}{4} + \frac{g^{s,xy}_{ij}}{2}\frac{\Delta^{v*}_{ij\downarrow}}{\Delta^{v*}_{ij\uparrow}}\Big)\Delta^{v*}_{ij\uparrow} + \frac{\partial W}{\partial g^{s,z}_{ij}}\frac{\partial g^{s,z}_{ij}}{\partial \Delta^v_{ij\uparrow}} \tag{S5}$$

and the effective local chemical potential is defined as

$$\mu_i = -\frac{\partial W}{\partial n_{i\sigma}} = \mu - \frac{1}{2}\sigma\sum_j g^{s,z}_{ij} J m^v_j - \sum_j \frac{\partial W}{\partial g^{s,xy}_{ij}}\frac{\partial g^{s,xy}_{ij}}{\partial n_{i\sigma}} - \sum_j \frac{\partial W}{\partial g^{s,z}_{ij}}\frac{\partial g^{s,z}_{ij}}{\partial n_{i\sigma}} - \sum_{j\sigma'} \frac{\partial W}{\partial g^t_{ij\sigma'}}\frac{\partial g^t_{ij\sigma'}}{\partial n_{i\sigma}} \tag{S6}$$

The last term $\partial g^t_{ij\sigma'}/\partial n_{i\sigma}$ in the effective local chemical potential gives the biggest contribution. This was not included in previous works using GWA to study CB[3,4], and their results have different patterns compared with ours. In addition the energy variation between different charge-ordered states is much larger than our nearly degenerate results.

Now H_{MF} can be rewritten as BdG equations,

$$H_{MF} = \left(c_{i\uparrow}^{\dagger}, c_{i\downarrow}\right) \begin{pmatrix} H_{ij\uparrow} & D_{ij} \\ D_{ji}^{*} & -H_{ji\downarrow} \end{pmatrix} \begin{pmatrix} c_{j\uparrow} \\ c_{j\downarrow}^{\dagger} \end{pmatrix} \tag{S7}$$

We can diagonalize the H_{MF} to obtain equal number of positive and negative eigenvalues with their corresponding eigenvectors (u_i^n, v_i^n). With these eigenvectors, we can determine the order parameters at zero temperature by following equations

$$\begin{aligned} n_{i\uparrow} &= \langle c_{i\uparrow}^{\dagger} c_{i\uparrow} \rangle_0 = \sum_{n_-} |u_i^n|^2 \\ n_{i\downarrow} &= \langle c_{i\downarrow}^{\dagger} c_{i\downarrow} \rangle_0 = \sum_{n_+} |v_i^n|^2 \\ \Delta_{ij\uparrow}^{v} &= \langle c_{i\uparrow} c_{j\downarrow} \rangle_0 = \sum_{n_+} u_i^n v_j^{n*} \\ \Delta_{ij\downarrow}^{v} &= -\langle c_{i\downarrow} c_{j\uparrow} \rangle_0 = \sum_{n_+} u_j^n v_i^{n*} \\ \chi_{ij\uparrow}^{v} &= \langle c_{i\uparrow}^{\dagger} c_{j\uparrow} \rangle_0 = \sum_{n_-} u_j^n u_i^{n*} \\ \chi_{ij\downarrow}^{v} &= \langle c_{i\downarrow}^{\dagger} c_{j\downarrow} \rangle_0 = \sum_{n_+} v_i^n v_j^{n*} \end{aligned} \tag{S8}$$

The sum for $n_+(n_-)$ means the set of eigenvectors with positive(negative) energies. An iterative method is used to solve H_{MF} self-consistently. The convergence is achieved for every order parameter if its value changes less than 10^{-3} between successive iterations. After the self-consistency is achieved, we calculate order parameters, their formula are

$$\begin{aligned} \Delta_i =& \sum_{\sigma} (g_{i,\sigma}^{t} g_{i+\hat{x},\bar{\sigma}}^{t} \Delta_{i,i+\hat{x},\sigma}^{v} + g_{i,\sigma}^{t} g_{i-\hat{x},\bar{\sigma}}^{t} \Delta_{i,i-\hat{x},\sigma}^{v} - g_{i,\sigma}^{t} g_{i+\hat{y},\bar{\sigma}}^{t} \Delta_{i,i+\hat{y},\sigma}^{v} - g_{i,\sigma}^{t} g_{i-\hat{y},\bar{\sigma}}^{t} \Delta_{i,i-\hat{y},\sigma}^{v})/8, \\ m_i =& \left(\sqrt{g_{i,i+\hat{x}}^{s,z}} + \sqrt{g_{i,i-\hat{x}}^{s,z}} + \sqrt{g_{i,i+\hat{y}}^{s,z}} + \sqrt{g_{i,i-\hat{y}}^{s,z}}\right) m_i^{v}/4, \\ K_{i,i+\hat{x}} =& \frac{1}{2} \sum_{\sigma} g_{i,i+\hat{x},\sigma}^{t} \langle c_{i\sigma}^{\dagger} c_{i+\hat{x}\sigma} \rangle + g_{i+\hat{x},i,\sigma}^{t} \langle c_{i+\hat{x}\sigma}^{\dagger} c_{i\sigma} \rangle, \\ K_{i,i+\hat{y}} =& \frac{1}{2} \sum_{\sigma} g_{i,i+\hat{y},\sigma}^{t} \langle c_{i\sigma}^{\dagger} c_{i+\hat{y}\sigma} \rangle + g_{i+\hat{y},i,\sigma}^{t} \langle c_{i+\hat{y}\sigma}^{\dagger} c_{i\sigma} \rangle, \\ K_i =& (K_{i,i+\hat{x}} + K_{i,i-\hat{x}} + K_{i,i+\hat{y}} + K_{i,i-\hat{y}})/4 \end{aligned} \tag{S9}$$

The values for the above quantities was shown in Table 2 of the main text for a typical AP-CDW stripe. Here we show the values for two other stripes in Table 1 and three CB patterns in Table 2.

A schematic illustration of CB like patterns is shown in Figure 1. Definitions of symbols are same as Figure 1 in the main text. Again, same as stripes shown in Figure 1 from the main text, we have the maximum hole density at sites either on the domain walls of AFM or pair field if AFM is absent. The latter part is different from previous results using GWA to study CB[3,4].

We can also examine the symmetry of bond orders, $K_{i,i+\hat{x}}$ and $K_{i,i+\hat{y}}$, as we did in Figure 5 of the main text by examining the Fourier transform. CB in Figure 3 shows clearly it can be thought of as the linear combination of stripes in x and y direction. The small dots inside the dotted square or the first Brillouin zone is proportional to the s' form factor or $A_{S'}$ discussed in the main text, and the outside dots are related to the d-form factor or A_D. Just like AP-CDW stripe, the AP-cCB in Figure 3c also has a much larger ratio of d/s'. For IP-CDW-SDW stripe and IP-cCB-sCB, the ratio is less than one. For AP-CDW-SDW and AP-cCB-sCB, the ratio is about 1.

We can also use the BdG solutions to calculate LDOS as shown in Figure 4 of the main text for the nPDW stripe. Here we use the supercell method[5] to calculate LDOS. Each cell has $N_x \times N_y$ lattice points and we have $M_c = M_x \times M_y$ cells. We can now reduce the Hamiltonian from $2M_xN_x \times 2M_yN_y$ to $M_x \times M_y$ matrix equations each with dimension $2N_x \times 2N_y$. LDOS is calculated by the equation

$$\rho_i(E) = \frac{1}{M_c} \sum_{\mathbf{K},n} [(g_{i\uparrow}^{t})^2 |u_i^n(\mathbf{K})|^2 \delta(E - E_n(\mathbf{K})) + (g_{i\downarrow}^{t})^2 |v_i^n(\mathbf{K})|^2 \delta(E + E_n(\mathbf{K}))] \tag{S10}$$

where $\mathbf{K} = 2\pi(\frac{n_x}{M_xN_x}, \frac{n_y}{M_yN_y})$, $n_x \in [0, M_x - 1]$ and $n_y \in [0, M_y - 1]$. Also we replace the delta function by a Lorentzian function with the width set to be 0.01t in this paper. In Figure 4 of the main text, we had used $M_x = M_y = N_x = N_y = 16$.

Reference

1. Zhang, F. C., Gros, C., Rice, T. M., & Shiba, H.. A renormalised Hamiltonian approach to a resonant valence bond wavefunction. *Supercond. Sci. Technol.* **1**, 36 (1988).
2. Yang, K., Chen, W., Rice, T. M., Sigrist, M. & Zhang, F. C.. Nature of stripes in the generalized $t-J$ model applied to the cuprate superconductors. *New J. Phys.* **11**, 055053 (2009).
3. Huang, H., Li, Y., & Zhang, F. C.. Charge-ordered resonating valence bond states in doped cuprates. *Phys. Rev. B* **71**, 184514 (2005).
4. Poilblanc, D.. Stability of inhomogeneous superstructures from renormalized mean-field theory of the $t-J$ model. *Phys. Rev. B* **72**, 060508 (2005).
5. Schmid, M., Anderson, B., Kampf, A., & Hirschfeld, P. J.. d-Wave Superconductivity as a Catalyst for Antiferromagnetism in Underdoped Cuprates. *New J. Phys.* **12**, 053043(2010).

IP-CDW-SDW

site number	1	2	3	4
δ_i	0.1141	0.1252	0.1369	0.1252
$\|m_i\|$	0.161	0.1146	0	0.1146
Δ_i	0.0255	0.0256	0.0256	0.0256
K_i	0.0771	0.0844	0.0925	0.0844
$K_{i,i+\hat{y}}$	0.0723	0.0856	0.1003	0.0856
$K_{i,i+\hat{x}}$	0.0818	0.0846	0.0846	0.0818

AP-CDW-SDW

site number	1	2	3	4
δ_i	0.1071	0.1268	0.1396	0.1268
$\|m_i\|$	0.2315	0.1189	0	0.1189
Δ_i	0	0.0219	0.0273	0.0219
K_i	0.0726	0.0844	0.0926	0.0844
$K_{i,i+\hat{y}}$	0.0971	0.0986	0.0927	0.0986
$K_{i,i+\hat{x}}$	0.048	0.0924	0.0924	0.048

Table 1. Values of several order parameters for IP-CDW-SDW and AP-CDW-SDW stripes at 0.125 doping.

IP-cCB-sCB

δ_i	1	2	3	4
1	0.0318	0.0594	0.1037	0.0594
2	0.0594	0.0849	0.1205	0.0849
3	0.1037	0.1205	0.1378	0.1205
4	0.0594	0.0849	0.1205	0.0849

Δ_i	1	2	3	4
1	0.0164	0.0162	0.0157	0.0162
2	0.0162	0.0167	0.0172	0.0167
3	0.0157	0.0172	0.0189	0.0172
4	0.0162	0.0167	0.0172	0.0167

$\|m_i\|$	1	2	3	4
1	0.3614	0.2879	0	0.2879
2	0.2879	0.2271	0	0.2271
3	0	0	0	0
4	0.2879	0.2271	0	0.2271

K_i	1	2	3	4
1	0.0216	0.0403	0.0683	0.0403
2	0.0403	0.0567	0.0793	0.0567
3	0.0683	0.0793	0.0933	0.0793
4	0.0403	0.0567	0.0793	0.0567

AP-cCB-sCB

δ_i	1	2	3	4
1	0.0686	0.083	0.1106	0.083
2	0.083	0.0959	0.1176	0.0959
3	0.1106	0.1176	0.1257	0.1176
4	0.083	0.0959	0.1176	0.0959

Δ_i	1	2	3	4
1	0	0	0	0
2	0	0.0051	0.0042	0.0051
3	0	0.0042	0.0055	0.0042
4	0	0.0051	0.0042	0.0051

$\|m_i\|$	1	2	3	4
1	0.3793	0.2857	0	0.2857
2	0.2857	0.221	0	0.221
3	0	0	0	0
4	0.2857	0.221	0	0.221

K_i	1	2	3	4
1	0.0464	0.0573	0.0757	0.0573
2	0.0573	0.0671	0.0819	0.0671
3	0.0757	0.0819	0.0906	0.0819
4	0.0573	0.0671	0.0819	0.0671

AP-cCB

δ_i	1	2	3	4
1	0.1229	0.1109	0.0926	0.1109
2	0.1109	0.1039	0.0884	0.1039
3	0.0926	0.0884	0.078	0.0884
4	0.1109	0.1039	0.0884	0.1039

K_i	1	2	3	4
1	0.0865	0.0786	0.0649	0.0786
2	0.0786	0.0717	0.0601	0.0717
3	0.0649	0.0601	0.0518	0.0601
4	0.0786	0.0717	0.0601	0.0717

Δ_i	1	2	3	4
1	0	0	0	0
2	0	0.0154	0.0198	0.0154
3	0	0.0198	0.0252	0.0198
4	0	0.0154	0.0198	0.0154

Table 2. Values of several order parameters for checkerboard patterns. The hole concentration is 0.1 for AP-cCB-sCB and AP-cCB but 0.09 for IP-cCB-sCB.

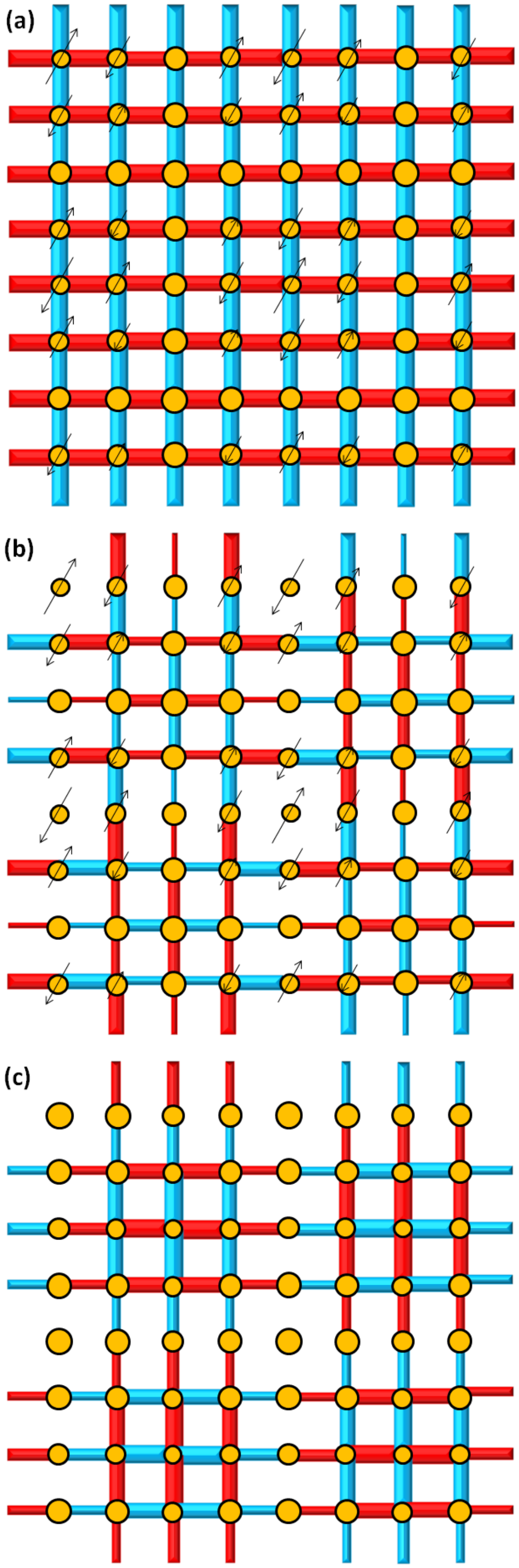

Figure 1. Schematic illustration of modulations for CB like patterns: (a)IP-cCB-sCB (b)AP-cCB-sCB (c)AP-cCB respectively. Definitions of all symbols are same as Figure 2 of the main text. The average hole density is 0.1 for AP-cCB-sCB and AP-cCB and 0.09 for IP-cCB-sCB.

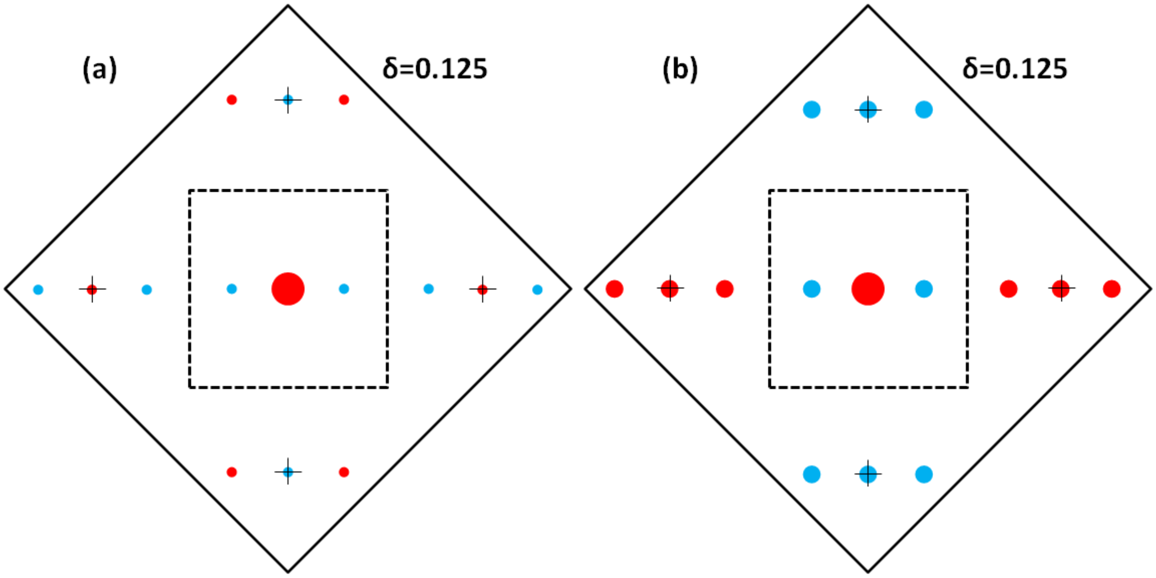

Figure 2. Schematic illustration of the Fourier transform of the bond orders for (a)IP-CDW-SDW stripe and for (b)AP-CDW-SDW. The dot size scales with the magnitude and red (blue) for positive(negative) values. "+" signs are at the four reciprocal lattice vectors $(\pm 2\pi/a_0, 0)$ and $(0, \pm 2\pi/a_0)$ and their nearby medium size dots are shifted from them by $(\pm\pi/2a_0, 0)$. The center large dot is $Q = (0,0)$ and has two red small dots nearby at $(\pm\pi/2a_0, 0)$. The inner dotted square is the boundary of first Brillouin zone. The doping for both stripes is 1/8.

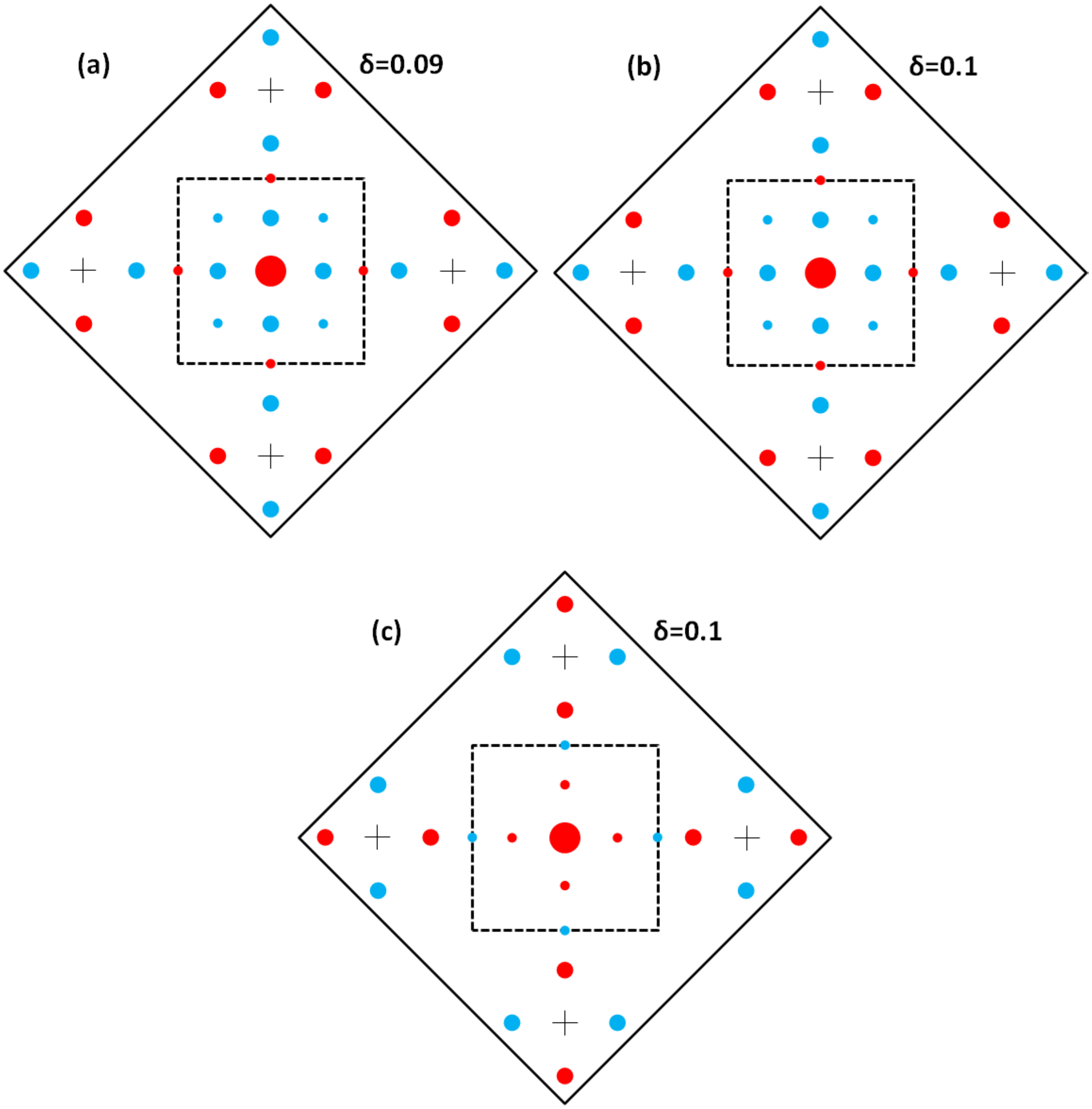

Figure 3. Schematic illustration of the Fourier transform of the bond orders for CB patterns (a)IP-cCB-sCB, (b)AP-cCB-sCB, and (c)AP-cCB. The hole density is (a)$\delta = 0.09$, (b)$\delta = 0.1$, and (c)$\delta = 0.1$. All the dots are shifted from $Q = (0,0)$ and the four reciprocal lattice vectors (denoted by "+" sign) by $(\pm 2\pi/a_0, 0)$ or $(0, \pm 2\pi/a_0)$. The notations are the same as those in Figure 2.
